# A co-designed intervention to support people living with achalasia to eat in a social setting: a feasibility study

**DOI:** 10.1186/s40814-024-01574-5

**Published:** 2024-12-19

**Authors:** Melika Kalantari, Amelia Hollywood, Rosemary Lim, Majid Hashemi

**Affiliations:** 1 School of Pharmacy, University of Reading, Reading, UK; 2 University College London Hospitals NHS Foundation Trust, London, UK

**Keywords:** Achalasia, Feasibility study, Co-design, APEASE, Intervention, Behaviour change, Eating behaviour, Chronic condition, Rare condition

## Abstract

**Background:**

Achalasia is a rare oesophageal condition that can affect eating behaviours. This study aimed to evaluate the feasibility of recruitment and assess the acceptability of a co-designed, workbook-based intervention targeting one of the most challenging eating behaviours, which was eating in a social setting.

**Methods:**

A mixed-method approach was employed, which involved pre- and post-intervention questionnaires and semi-structured interviews. The Achalasia Action group, a UK-based support group, facilitated participant recruitment. The intervention was a workbook designed collaboratively by the researchers and people living with achalasia, with strategies built on the COM-B model (Capability, Opportunity, Motivation-Behaviour). Outcome measures were based on recruitment and retention rates, the APEASE criteria for usability and acceptability, self-reported changes in eating behaviours, and qualitative feedback from participant interviews.

**Results:**

The study aimed to recruit 20 participants, and this target was achieved, resulting in a 100% recruitment rate. However, the post-intervention questionnaires were completed by only 10 participants, indicating a 50% retention rate from baseline. No issues were raised with completing the pre- and post-questionnaires, from completers. The quantitative feedback from participants indicated that they found the workbook activities clear, easy to understand, and complete, with the majority reporting positive experiences. Qualitative feedback on the intervention described enhanced social support and improved symptom management of achalasia in a social setting. Furthermore, the intervention met the APEASE criteria, indicating its usability and acceptability.

**Conclusions:**

This study explored the feasibility of recruiting and retaining people living with achalasia in intervention research, highlighting the acceptability of the co-designed intervention to improve social eating experiences. However, with a retention rate of only 50% at follow-up, it is evident that future studies should explore the reason behind this and also consider recruiting a larger baseline sample to ensure the target is achieved. The positive outcomes of the co-designed intervention underscore the importance of user involvement in developing interventions. The intervention demonstrated the potential to support people living with achalasia in eating in a social setting. The co-designed intervention has significant practical implications by providing healthcare professionals and support groups with a feasible, potentially effective method to enhance the social eating experience of people living with achalasia, potentially improving their overall quality of life.

## Key messages regarding feasibility

1. *What uncertainties existed regarding the feasibility?* At the outset of the study, there were uncertainties related to the feasibility of recruitment and retention of individuals living with achalasia in intervention research. These uncertainties stemmed from concerns about whether an adequate number of participants could be recruited and whether individuals living with achalasia would be willing and able to participate adequately in the study.
2. *What are the key feasibility findings?* The study achieved its aim of recruiting the target sample size of participants living with achalasia, addressing one of the initial uncertainties. However, it was observed that the retention rate at follow-up was only 50%, indicating challenges in maintaining participant engagement over time. Importantly, the co-designed intervention, which involved the active participation of individuals living with achalasia, yielded positive outcomes, showing promise in improving the social eating experiences of this population.
3. *What are the implications of the feasibility findings for the design of the main study?* The feasibility findings have important implications for the design of future studies. To address the challenges in participant retention, it is recommended that future studies consider not only recruiting a larger baseline sample to ensure a sufficient number of participants at follow-up for the main study but also arranging a patient group meeting in order to address the issue of retention. Additionally, the potential feasibility of the co-designed intervention underscores the importance of involving users in the development of interventions, as this approach not only enhances feasibility but also can lead to more effective interventions. The co-designed intervention has practical implications for healthcare professionals and support groups, providing a feasible method to enhance the social eating experience and overall quality of life for individuals living with achalasia.

## Background

Achalasia is defined as an uncommon, chronic condition that affects the motility of the oesophageal body along with altered lower oesophageal sphincter (LOS) relaxation. [1] The symptoms of achalasia, including dysphagia, regurgitation, chest pain, weight loss, and occasional vomiting, disrupt patients’ ability to eat, socialise, and maintain their physical and emotional well-being. This combination of symptoms often leads to significant challenges in daily life, impacting both the individual’s physical health and psychological state. [2] Even the most effective treatments are unlikely to be curative. A multidisciplinary team, including gastroenterologist, surgeon, radiologist, and dietician, are needed to obtain optimal outcomes for managing this rare chronic condition. The main goal of medical treatments and interventions is mitigation of symptoms. The medical interventions are pharmacologic, endoscopic, and surgical treatments to achieve symptom relief. [3] As all medical treatments only help to alleviate symptoms, it is important for people living with achalasia to use non-pharmacological interventions to manage their condition.

In recent years, research has yielded promising findings on the effectiveness of self-help protocols delivered by audio/videotapes, brochures, and manuals in addressing mental health and substance use problems. [4] While these findings are not specific to achalasia, they suggest the potential of similar self-help approaches in the management of long-term health conditions. The evidence in the current literature also shows the benefits of nonmedical intervention on different chronic conditions. For example, a study carried out by Pujol et al. discussed the importance of non-pharmacological intervention, such as physiotherapy in adjunction with medical treatments for patients with cancer pain. The study also stressed the significance of attending to psychological issues such as affective distress, coping, and beliefs about cancer as a crucial aspect of pain treatment programmes. Furthermore, psychophysiological interventions such as biofeedback and relaxation were employed as behavioural strategies. These interventions have been found to reduce pain and enhance patients’ quality of life. [5] Similarly, Ambrose et al. highlighted the importance of non-pharmacological interventions in treating chronic pain. These interventions can provide an alternative or complementary approach to traditional pharmacological treatments. Given the modest relief and high discontinuation rates associated with pharmacological treatments due to adverse effects, these non-pharmacological interventions are invaluable. [6] One of the examples is cognitive behavioural therapy which uses behaviour change strategies to reduce pain and fatigue. Other non-pharmacological interventions such as acupuncture, mindfulness meditation, yoga, and relaxation have also become accepted forms of symptom management, with clinical trials demonstrating efficacy for pain and physical function. [6]

Based on a study carried out by Kalantari et al. [7] exploring the experiences of people living with achalasia, self-management was a common approach people used to manage their long-term condition. [7] While a range of medical treatment options exist for people living with achalasia, these alone are often insufficient to manage the condition. Therefore, people living with achalasia often have to adopt additional techniques to alleviate symptoms or cope with new symptoms that may arise as a result of the medical treatments themselves. This emphasises the important role that people living with achalasia play in managing their long-term condition, particularly in terms of modifying daily activities, such as eating, to alleviate symptoms. Research has indicated that stress can alter eating patterns, affecting the types, quantities, and variety of food consumed. [8] Stress not only influences an individual’s health behaviours but also their reactions to stressors, such as changes in eating habits. Such deviations, especially when faced with chronic stress and the challenges of managing long-term conditions, may heighten the risk of developing disordered eating behaviours. [9]

Eating disorder symptoms are linked to major problems with mental and physical health, can last for a long time, and can lead to clinical eating disorders, which are linked to substantial incidents of illness and death. [10] Therefore, prompt intervention significantly improves the exacerbation of symptoms. Self-help interventions have been suggested as the primary course of action for addressing mild to moderate symptoms of eating disorders. [11] Self-help interventions are structured programmes that people can work through on their own or with little help. They include tasks and activities based on evidence and theory. [12] These kinds of interventions are scalable; can give users privacy, easy access, and a lot of freedom; and are suggested for mild to moderate eating disorder symptoms. [13, 14]

There are interventions that promote supported self-management and can improve long-term outcomes by providing individuals with skills and information for them to manage chronic conditions effectively. [15] It is debated whether interventions to change behaviour should have a strong theoretical background to promote change. However, interventions based on behaviour change theory in certain long-term conditions, such as rheumatoid arthritis and lower back pain, have shown the potential to improve long-term behaviour. [16] In a comprehensive meta-analysis and systematic review conducted by Cradock et al., focused on dietary behaviour modification techniques implemented in type 2 diabetes management, a subset of four distinct BCTs were pinpointed as efficacious in reductions in HbA1c (a marker of long-term blood sugar control). These encapsulated techniques of problem-solving, provision of feedback on behaviour, integration of objects into the environment, social comparison, and application of relevant theoretical frameworks. [17]

Research highlights the importance of supporting self-management of long-term conditions, and this can be achieved through interventions based on theory and evidence. Kalantari et al. [7] conducted an in-depth examination of the journey experienced by people living with achalasia, elucidating the sequential steps involved and identifying areas necessitating additional support. [7] Based on the data collected in the initial study and the insights gained from it, eating behaviour was the main concern raised; therefore, a subsequent study was conducted to address this. Kalantari et al. (2023) employed a co-design approach to identify a specific eating behaviour and develop an intervention in collaboration with people living with achalasia. The specific challenge that was identified was eating in a social setting such as eating with other people or in a public place, which is a primary concern faced daily by people living with achalasia. [18] Informed by the collaboration between researchers and people living with achalasia, as well as grounded in scientific evidence and the Theoretical Domains Framework (TDF), the content of the intervention was co-designed. A self-directed workbook was confirmed as the appropriate mode of delivery, and it was iteratively developed by participants and researchers.

Informed by these previous studies, the aim of the current study was to explore the feasibility of recruitment and testing the acceptability of the intervention in supporting people living with achalasia in social settings. By evaluating its efficacy, we sought to investigate the extent to which the co-designed intervention could facilitate desired behavioural changes in people living with achalasia. This study also explored the practical aspects of recruitment and evaluating the feasibility of enrolling participants for the intervention evaluation. By examining both the feasibility of recruitment and testing the acceptability of the intervention, we aimed to provide valuable insights into the viability and potential impact of implementing the co-designed intervention within real-world settings.

### Aim and objectives

The purpose of feasibility studies is to determine whether further testing of an intervention is justified; they allow researchers to determine whether or not the ideas and findings can be made relevant and sustainable. A feasibility study evaluates the practicability of a study, examining its achievability, potential value, and optimal implementation strategies. [19] Such research may identify what needs to be modified in the research methods or protocols and how such modifications may be implemented. [20] Therefore, this study’s primary aim is to assess the feasibility of undertaking a study evaluating a novel co-designed intervention to support people living with achalasia to eat in a social setting. Key study objectives were informed by existing feasibility guidelines [10, 20] and are to (1) explore the feasibility of recruiting and retaining participants in the study, (2) determine the acceptability of measures and research procedures, and (3) conduct a mixed -methods process evaluation to determine the acceptability of the intervention to participants.

## Methods

### Design

The study employed a mixed-methods approach, utilising both pre- and post-intervention questionnaires and semi-structured interviews. The questionnaires were used to quantitatively assess changes in eating behaviours and achalasia symptoms along with gathering feedback on the usability and design of the workbook. Semi-structured interviews provided qualitative insights into the participants’ experiences and perspectives, further aiding in the evaluation of the feasibility and potential acceptability of the co-designed intervention. A favourable ethical opinion was granted through the University of Reading School of Chemistry, Food and Pharmacy Research Ethics Committee (SREC 51/2022).

### Sample size

In determining the sample size for this study, several factors influenced the decision to include 20 participants. The sample size of 20 participants was chosen to align with the primary objectives of this feasibility study. Firstly, given the exploratory nature of the research, a smaller cohort was deemed appropriate, allowing for an in-depth examination of individual experiences and perspectives. According to research guidelines, a sample size of 20–30 is generally deemed acceptable for pilot and feasibility studies, as it allows for a manageable and thorough evaluation of study logistics and feasibility without overextending resources. [21] The specific criteria and rarity of achalasia further limited the pool of potential participants. However, this sample size was considered sufficient to provide a reasonable estimate of key parameters, such as the standard deviation of primary outcomes and participant retention rates, with a degree of precision that would inform future studies. Moreover, empirical studies further support this sample range for feasibility research, suggesting that sample sizes within 20–30 participants are often sufficient for obtaining reliable preliminary insights. Specifically, qualitative research on saturation indicates that new themes tend to emerge within the first 12–17 interviews, suggesting that smaller samples can provide robust, foundational insights. [22] This sample size also allowed for a mixed-methods process evaluation to determine how acceptable the intervention was to participants, capturing a range of experiences and perspectives. In addition, constraints related to time and resources were a factor. Taking all these considerations into account, a sample size of 20 was determined to be both suitable and manageable for the study’s objectives. Insights gained from the recruitment methods and the rate of participant enrolment observed in this study will inform the planning and feasibility considerations for a larger sample size in a future definitive trial, ensuring more robust recruitment and retention strategies.

### Procedure

The study involved assessing the feasibility and potential acceptability of the behaviour change intervention. Baseline measures included a quantitative pre-intervention questionnaire that collected data on participants’ demographics and eating behaviour. Participants were asked to complete the pre-intervention questionnaire before they attempted to use the workbook. The implementation of the intervention then followed, where participants were introduced to the workbook and had the opportunity to put its contents into practice. After completing the workbook, participants completed a post-intervention questionnaire, which assessed current eating behaviour and beliefs, along with the design and usability of the workbook. Subsequently, an online one-to-one interview was conducted to gather in-depth feedback and personal insights regarding participants’ experiences with the workbook and the intervention as a whole, using the APEASE criteria. The APEASE criteria can be used by intervention designers to identify the intervention functions, policy categories, behaviour change strategies, and delivery methods that are most suitable for their context and, therefore, most likely to be implemented and have an impact. APEASE is Affordability, Practicality, Effectiveness and cost-effectiveness, Acceptability, Side effects/safety, and Equity of the intervention. [23]

### Recruitment

Recruitment was facilitated by Achalasia Action, which is an independent charity supporting people living with achalasia in the United Kingdom (UK). The researcher emailed the study recruitment letter and information sheet to the administrator of Achalasia Action, who then sent these onto their members using a mailing list. [24] At that time, the group had approximately 300 active members. According to the administrator of the Achalasia Action group, 30 participants requested further information on the study. Despite the outreach, several potential participants could not join due to various reasons, including not being UK residents and opting to assist with another study running at the same time or their participation in related earlier studies. [7, 18] Consequently, the pool of eligible participants was reduced.

The inclusion criteria for this study were as follows: anyone who lives in the UK, aged 18 years or over, has a confirmed diagnosis of achalasia (self-reported), can complete or attempt to complete the co-designed workbook, had not taken part in the previous co-design study which developed the intervention under evaluation, can access the internet for communication via Microsoft Teams, and can read, speak, and understand English. Proficiency in English and access to Microsoft Teams are essential to ensure effective communication and participation in virtual study activities, thereby facilitating robust engagement and accurate comprehension of study materials. The recruitment was active for 91 days over 14 weeks, to achieve the required sample size.

Participants who registered their interest to take part in this study received the participation information sheet and a link to the online consent form by email, which was hosted on JISC Online Surveys. [25] The information sheet explained the voluntary nature of the study and the potential risks and benefits. The recruitment stopped when the target number of participants (20 participants) completed the baseline measures.

### Data collection

Participants were sent the pre-intervention questionnaire in November 2022, and they were given up to 2 months to complete the questionnaire and attempt to use the workbook. They were then asked to complete the post-intervention questionnaire in January 2023 and participate in an interview in February 2023. Participants were contacted 4 weeks after receiving the workbook (December 2022), 8 weeks later (February 2022), and 12 weeks later (March 2023) to be reminded to complete the post-intervention questionnaire. Participants who did not respond to these follow-up attempts were grouped as lost to follow-up. The administrator, who assisted with participant recruitment, suggested several potential reasons for noncompletion, including forgetfulness, reluctance to discuss personal issues, health problems, and the added stress of the COVID-19 pandemic at that time. It is important to note that while these participants may have used the intervention, their lack of response to follow-up attempts prevented the researcher from obtaining their insights, underscoring the distinction between completing the intervention study and completing the intervention itself.

After completing the consent form, participants were sent a link by email to complete the online demographic questionnaire along with the pre-intervention questionnaire, also hosted on JISC Online Surveys. Once the demographic details and the pre-intervention questionnaire were completed, participants were emailed a printable version of the intervention, i.e. the workbook. Participants were asked to print the workbook or contact the researcher to receive a printed copy by post. Out of the 20 participants, 16 received the intervention via email, 4 received it by post. Among completers and non-completers, four completers received the intervention by post, while five non-completers received it by post. Participants were given up to 2 months to complete the workbook. Participants indicated they had finished the workbook by emailing the researcher and were then sent a link to the post-intervention questionnaire. Participants who completed the post-intervention questionnaire were asked to indicate whether they were interested in taking part in a 30-min one-to-one online semi-structured interview using Microsoft Teams (an online meeting platform). Even though non-completers were contacted on numerous occasions, no response was received from them. Therefore, it cannot be ascertained whether these participants completed the intervention but declined to complete the post-intervention questionnaire.

### Pre-intervention questionnaire

The pre-intervention questionnaire included questions on the demographic details of the participants in order to describe the sample in the current study. It also asked participants questions about the number of times in which they ate in a social setting in a set period of time, the level of enjoyment and confidence when eating in a social setting, and the symptoms experienced when eating in a social setting. There are no validated measures of symptoms for achalasia; therefore, the measures used in the current study were based on symptoms reported by people living with achalasia highlighted in a previous study. [18] Participants were asked to grade their symptoms of pain, heartburn, and regurgitation on a scale from 1 to 5, with 1 experiencing no symptoms and 5 having severe symptoms. This self-report scale provided a standardised measure of symptom intensity for each individual. The aim of these questions was to explore the impact of using the intervention workbook.

### The intervention workbook

The theory-based intervention comprised a workbook with three sections, co-designed by the researchers and people living with achalasia. The workbook, presented in the English language, comprised 29 pages and was formatted in A5 size. The content of the workbook was not assessed for reading level as the inclusion criteria for this study were for people who self-reported that they can read, speak, and understand English. The content of the intervention was developed using the behaviour change wheel (BCW), which applies the COM-B model (Capability, Opportunity, and Motivation-Behaviour) and the Theoretical Domains Framework (TDF). Each chapter started with quotes from other people living with achalasia, sourced from the co-design study carried out by Kalantari et al. (2023). [18] These quotes provided real-life perspectives on the experiences and challenges faced by those with the condition, particularly focusing on the primary target behaviour: eating in a social setting. For instance, one quote stated, “I accept my condition and try to eat what I can eat confidently”. The aim of the quotes was to give ideas to people using the workbook to explore and try different options and activities in order to enhance the eating experience in a social setting. The sections then introduced various activities and techniques to focus on the target behaviour. For instance, the workbook guided participants through goal-setting for comfortable eating in public, planning and implementing changes in their approach, and strategies to reduce negative emotions associated with eating socially. These components collectively aimed to equip individuals with tools and coping strategies to improve their comfort and confidence in social setting situations.

Participants were given instructions on how to use the workbook. They were provided additional spaces to add any further comments or feedback on the content and design of the workbook in order to discuss with the researcher in the one-to-one interview.

### Post-intervention questionnaire

The post-intervention questionnaire included repeated eating behaviour measures, i.e. similar questions to the pre-intervention questionnaire, as the aim of the questionnaires was to compare the data before and after using the workbook. The questionnaire also included a series of feedback questions on the content of the workbook and its usability and practicality. Participants were then asked whether they wanted to take part in an interview to share further feedback on the workbook.

### Online interviews

Upon completion of the post-intervention questionnaire, participants were invited to take part in an online interview to share their personal experiences and thoughts related to the workbook. Conducted via Microsoft Teams, these interviews followed a semi-structured format, which allowed for flexibility in discussion and ensured core topics were addressed. The interview protocol was designed to explore participant perspectives on the content, design, and usability of the workbook, as well as the perceived practicability of the intervention. The interviews also provided a platform for participants to voice any potential challenges or barriers they encountered while engaging with the intervention. The detailed qualitative insights derived from these interviews were crucial in further assessing the feasibility and potential impact of the intervention in a real-world context.

### Data analysis

The quantitative data gathered from pre- and post-intervention questionnaires were subjected to descriptive statistical analysis. This provided a summary of the central tendency, dispersion, and distribution patterns of the participants’ responses. The feasibility of the workbook was assessed to determine its practicality and usability. Quantitative data gathered from the questionnaires provided valuable insights into changes in eating behaviours, achalasia symptoms, and feedback on the workbook’s usability and design. The changes in participants’ behaviours and related symptoms were quantified using a Likert scale ranging from 1 (not at all severe) to 5 (very severe), to measure the potential feasibility of the intervention. The significance of the changes could not be tested due to the sample size, and this was not the primary aim of the study.

In parallel, for the qualitative data drawn from the post-intervention interviews, the APEASE criteria was utilised (Acceptability, Practicability, Effectiveness, Affordability, Safety, and Equity) as a guiding framework. This approach helped us systematically categorise participants’ feedback and experiences, giving us rich, contextualised insights into their perception of the intervention’s utility, design, delivery, and content. Taken together, this combined approach offered us a holistic understanding of the intervention’s impact and its potential for further implementation. The acceptability of the intervention was assessed through qualitative interviews, which elicited participants’ feedback on their experiences and perceptions. Practicability was evaluated by considering the feasibility and practicality of implementing the intervention in real-world settings. Potential effectiveness was examined by assessing changes in eating behaviours and related symptoms reported by participants. Affordability was not specifically analysed in this study; however, participants were asked whether they would be happy to print the workbook for future use and whether the cost of printing could be an issue for them. Safety was monitored throughout the intervention period, ensuring that no adverse effects or risks were encountered. Finally, equity was taken into account by considering the intervention’s potential applicability and benefits for people living with achalasia across diverse backgrounds. This evaluation method allowed for a more in-depth understanding of the user’s reaction to the intervention’s design, delivery, content, and influences on understanding and engagement.

In conjunction with the APEASE criteria and descriptive statistical analysis, the Capability, Opportunity, and Motivation-Behaviour (COM-B) model was used as a theoretical framework to guide the interpretation of the data. This model supported the deductive analysis of key factors influencing participants’ changes in behaviour as a result of the intervention. In the context of the study, “capability” refers to participants’ ability to implement the strategies proposed in the workbook, “opportunity” refers to the external conditions facilitating or hindering their engagement with the intervention, and “motivation” refers to the intrinsic and extrinsic processes that energise and direct their behaviour.

## Results

### Sample

The recruitment was active for 91 days over 14 weeks, and 21 eligible participants consented to take part in this study. Twenty people provided informed consent and completed the pre-intervention questionnaire. One participant completed the consent form but did not complete the pre-intervention questionnaire and therefore was omitted from the study. Interviews with the five participants lasted between 17 and 28 min (mean = 23.2 min). A total of 20 participants were recruited for the study. Ten participants were retained for both baseline and follow-up assessments, comprising the completer group, while the remaining 10 were categorised as non-completers, having solely completed baseline measures. This comparison aims to shed light on factors influencing study participation and engagement, although it is essential to note that the analysis remains descriptive in nature, lacking statistical inference.

Table 1 displays the demographic characteristics of participants across both groups. While both cohorts exhibited a diverse age distribution, a significant majority were aged over 54, with no substantial deviation between completers and non-completers. Female participants constituted the majority, 85% (*n* = 17), across both groups, indicating a consistent gender distribution. Among the 20 participants recruited for the study, various employment statuses were observed. Retirement emerged as the predominant category, with 45% (*n* = 9). Full-time employment constituted a significant subset of individuals, with 25% (*n* = 5) of participants dedicated to full-time work. Solely one participant 5% (*n* = 1) reported being unemployed, and a small percentage 5% (*n* = 1) of participants were classified under the “other” category.

**Table 1:** Demographic data of participants (*n* = 20)

Variable	Non-completers	Completers	Total
	**Demographics**		
**Number of participants**	**10**	**10**	**20**
**Age (years)** 18–24 25–34 35–44 45–54 Above 54	1 (10%) 0 2 (20%) 3 (30%) 4 (40%)	0 1 (10%) 2 (20%) 2 (20%) 5 (50%)	*n* = 1 (5%) *n* = 1 (5%) *n* = 4 (20%) *n* = 5 (25%) *n* = 9 (45%)
**Gender** Male Female	2 (20%) 8 (80%)	1 (10%) 9 (90%)	*n* = 3 (15%) *n* = 17 (85%)
**Employment status** Full-time Part-time Unemployed Retired Other	3 (30%) 3 (30%) 0 4 (40%) 0	2 (20%) 1 (10%) 1 (10%) 5 (50%) 1 (10%)	*n* = 5 (25%) *n* = 4 (20%) *n* = 1 (5%) *n* = 9 (45%) *n* = 1 (5%)

### Recruitment and retention

The study collected recruitment data to assess the viability of participant recruitment and determine the recruitment rate (N recruited ÷ recruitment time [weeks]). [26] The recruitment rate for this study is 1.5. The pre-intervention questionnaire was completed by 20 participants (20/21, 95% retention). Post-intervention questionnaires were completed by 10 participants (10/20, 50% retention), and 5 participants showed interest in participating in an interview after completing the post-intervention questionnaire and intervention (5/10, 50%).

### Baseline characteristics of participants

The baseline measures reveal varied experiences among the participants living with achalasia (Table 2). A large proportion of the participants 45% (*n* = 9) had been diagnosed with achalasia for over 5 years, with 40% (*n* = 8) having been diagnosed for over 1 year, and a smaller percentage 15% (*n* = 3) having been diagnosed for less than 6 months. Concerning strategies to aid eating in social settings, a significant portion of participants 75% (*n* = 15) had attempted various interventions, while 25% (*n* = 5) had not pursued any specific strategies. When considering the frequency of eating out, participants demonstrated diverse habits, with twice a month being the most common frequency 30% (*n* = 6), followed by three times a month 20% (*n* = 4). Relatively fewer participants reported eating out twice a week 10% (*n* = 3) or six times a month 5% (*n* = 1). In terms of enjoying eating in social settings, 60% (*n* = 12) of participants reported currently enjoying it, while 30% (*n* = 6) did not, and 10% (*n* = 2) were uncertain.

**Table 2:** Baseline measures (*n* = 20)

Variable	**Non-completers**	**Completers**	**Total**
Number of participants	10	10	20
	**Demographics**		
**Length of time since diagnosis** < 6 months > 1 year > 5 years	3 (30%) 4 (40%) 3 (30%)	0 4 (40%) 6 (60%)	*n* = 3 (15%) *n* = 8 (40%) *n* = 9 (45%)
**Have they tried anything to help them eating in a social setting?** Yes No	7 (70%) 3 (30%)	8 (80%) 2 (20%)	*n* = 15 (75%) *n* = 5 (25%)
**How often do they eat out?** Very little if at all Twice a week Once a month Twice a month Three times a month Four times a month Six times a month	0 1 (10%) 1 (10%) 3 (30%) 4 (40%) 1 (10%) 0	1 (10%) 1 (10%) 2 (20%) 3 (30%) 0 2 (20%) 1 (10%)	*n* = 1 (5%) *n* = 2 (10%) *n* = 3 (15%) *n* = 6 (30%) *n* = 4 (20%) *n* = 3 (15%) *n* = 1 (5%)
**Do they currently enjoy eating in social setting?** Yes No Do not know	6 (60%) 3 (30%) 1 (10%)	6 (60%) 3 (30%) 1 (10%)	*n* = 12 (60%) *n* = 6 (30%) *n* = 2 (10%)

Table 3 presents the comparison of data collected for all 20 participants at baseline, irrespective of completion status. The scales used to measure each variable range from 1 (indicating the lowest level) to 5 (indicating the highest level). The variables assessed included enjoyment, confidence, pleasure, and various symptoms related to eating in a social setting. The questions relating to their experiences included the following: How confident are you in eating in a social setting? How much do you enjoy eating in a social setting? How pleasurable was the last time you ate in a social setting? These were on a scale from 1 (not at all) to 5 (very much). For enjoyment, both groups exhibited similar average scores, with a mean of 3.4 (*SD* = 1.09) across all participants. Likewise, no notable difference was observed in confidence levels, with an average score of 3.05 (*SD* = 1.04) for all participants. In terms of pleasure, participants reported an average score of 3.5 (*SD* = 1.27), indicating a moderate level of pleasure associated with eating in social settings.

The questionnaire asked participants to rate their symptom intensity with the following prompt: “Below are some common symptoms experienced by people living with achalasia. Please tick the level of the severity of the symptoms experienced when eating in a social setting over the past month on a scale of 1 to 5 (1 = Not at all, 5 = Very much). Over the past month in a social setting, I have felt…….”. Then a list of symptoms were provided for participants to score; these included pain, regurgitation, heartburn, nervousness, stress, and anxiety. These ratings reflect participants’ experiences and attitudes, specifically within social eating contexts. On average, participants reported a moderate level of pain (*M* = 2.55, *SD* = 1.07), regurgitation (*M* = 2.25, *SD* = 1.39), and heartburn (*M* = 1.93, *SD* = 1.18). Additionally, participants reported experiencing nervousness (*M* = 3, *SD* = 1.28), stress (*M* = 3.1, *SD* = 1.03), and anxiety (*M* = 3.25, *SD* = 1.19) to a moderate extent.

These findings suggest that participants, regardless of completion status, exhibited similar baseline characteristics in terms of enjoyment, confidence, pleasure, and symptoms related to eating in social settings. Further analysis is needed to explore the potential effectiveness of the intervention in modifying these baseline measures.

**Table 3:** Comparing the data collected for both completers and non-completers at baseline

Eating in a social setting	**Non-completers**		**Completers**		**Total**
	*n* = (range)	Mean (SD)	*n* = (range)	Mean (SD)	Mean (SD)
**Experiences**					
Enjoyment	*n* = 10 (2–4)	3.2 (0.92)	*n* = 10 (1–5)	3.6 (1.26)	3.4 (1.09)
Confidence	*n* = 10 (1–4)	2.7 (1.06)	*n* = 10 (2–4)	3.4 (0.84)	3.05 (1.04)
Pleasure	*n* = 10 (1–5)	3.2 (1.13)	*n* = 10 (1–5)	3.8 (1.40)	3.5 (1.27)
**Symptoms**					
Pain	*n* = 10 (2–4)	2.8 (0.79)	*n* = 10 (1–4)	2.3 (1.34)	2.55 (1.07)
Regurgitation	*n* = 9 (1–5)	3.4 (1.33)	*n* = 10 (1–4)	2.1 (1.45)	2.75 (1.39)
Heartburn	*n* = 8 (1–3)	1.87 (0.83)	*n* = 10 (1–5)	2 (1.41)	1.94 (1.18)
Nervous	*n* = 10 (1–5)	3.2 (1.32)	*n* = 10 (1–5)	2.8 (1.23)	3 (1.28)
Stressed	*n* = 10 (3–5)	3.7 (0.82)	*n* = 10 (1–5)	2.5 (1.18)	3.1 (1.03)
Anxious	*n* = 8 (3–5)	3.7 (0.89)	*n* = 10 (1–5)	2.8 (1.13)	3.24 (1.19)

In Table 4, the pre- and post-intervention data are presented, detailing participants’ experiences before and after the intervention across various variables. Post-intervention, there was a slight decrease in enjoyment (mean change of − 0.5) and pleasure (mean change of − 0.3) related to eating in social settings, alongside modest improvements in confidence (mean change of 0.2). However, these changes should be interpreted cautiously, given the considerable variability observed in participant responses, as indicated by the standard deviations. Similarly, changes in symptoms post-intervention varied, with some symptoms showing slight decreases (e.g. regurgitation, nervousness) but also exhibiting notable variability.

**Table 4:** Eating in a social setting and the level of pleasure, confidence, and enjoyment before and after the intervention (completers)

**Variable**	**Pre-intervention**		**Post-intervention**			**95% *CI***	**Descriptor**
	*n* = (range)	Mean (SD)	*n* = (range)	Mean (SD)	Mean change		
Enjoyment	*n* = 10 (1–5)	3.6 (1.26)	*n* = 10 (1–5)	3.1 (0.99)	− 0.5	(− 1.31, 0.31)	Positive change
Confidence	*n* = 10 (2–4)	3.4 (0.84)	*n* = 10 (2–5)	3.6 (0.97)	0.2	(− 0.45, 0.85)	Positive change
Pleasure	*n* = 10 (1–5)	3.8 (1.40)	*n* = 10 (2–5)	3.5 (0.97)	− 0.3	(− 1.16, 0.56)	Positive change
**Symptoms**							
Pain	*n* = 10 (1–4)	2.3 (1.34)	*n* = 10 (1–3)	2 (0.82)	− 0.3	(− 1.09, 0.49)	Negative change
Regurgitation	*n* = 10 (1–4)	2.1 (1.45)	*n* = 10 (1–4)	1.6 (1.07)	− 0.5	(− 1.41, 0.41)	Negative change
Heartburn	*n* = 10 (1–5)	2 (1.41)	*n* = 10 (1–4)	2 (1.05)	0	(− 0.88, 0.88)	No change
Nervous	*n* = 10 (1–5)	2.8 (1.23)	*n* = 10 (1–4)	1.8 (0.92)	− 1	(− 1.59, − 0.41)	Negative change
Stressed	*n* = 10 (1–5)	2.5 (1.18)	*n* = 10 (1–4)	1.6 (0.97)	− 0.9	(− 1.57, − 0.23)	Negative change
Anxious	*n* = 10 (1–5)	2.8 (1.13)	*n* = 10 (1–4)	1.8 (0.92)	− 1	(− 1.59, − 0.41)	Negative change

Qualitative data from interviews confirmed the questionnaire findings, indicating that the workbook intervention positively influenced participants’ eating behaviour in social settings. All five participants reported increased confidence and a shift in focus from finishing their meal to enjoying others’ company. Participants described the workbook as “very useful”, “informative”, and a “confidence builder”. According to the results of the pre- and post-questionnaire, which aimed to measure the impact of the workbook intervention on individuals’ eating behaviour in a social setting, three respondents reported a positive impact, four were uncertain, and three reported no impact.

### Usability of the intervention

Participants provided feedback on the clarity and level of difficulty of the workbook activities in the post-intervention questionnaire. Of the 10 participants who completed the workbook, 9 reported the activities easy to understand, with one expressing uncertainty. Similarly, when asked about the ease of completion of the workbook activities, eight participants responded positively, while two were uncertain. As part of the post-intervention questionnaire, participants were also asked about the impact of the workbook on their ability to enjoy and feel comfortable eating in a social setting. Out of the 10 participants, 6 reported a positive impact, 3 were unsure, and 1 reported a negative impact as they were unable to apply the activities of the workbook in many instances. Participants were asked about the potential efficacy of the workbook in assisting people living with achalasia at any stage following their diagnosis. Of the 10 participants, 7 answered positively, 2 reported a negative response, and 1 was unsure about the potential benefits of the workbook intervention. Among the two participants who reported a negative response, one mentioned that the workbook would be helpful to those who were newly diagnosed with achalasia, and the second participant said that when achalasia is severe, this workbook might be overwhelming or less beneficial. Participants were asked about their ability to complete the workbook activities independently in the post-intervention questionnaire. Nine confirmed their capability to do so, while one reported their inability to complete the workbook activities independently and noted that drawing on advice from others was helpful.

In the pre-intervention questionnaire, participants were asked to retrospectively assess the frequency of eating in a social setting over the past month. The same question was posed in the post-intervention questionnaire. Of the 10 completers, 4 participants reported an increase in eating in a social setting and an intention to increase their engagement in social setting occasions following their use of the workbook. Three participants reported no change in the frequency of eating in a social setting, while three others stated that the frequency in which they ate in a social setting had decreased after completing the intervention.

### APEASE

This section presents the findings from the feasibility assessment, narratively presented using the APEASE criteria. The data was obtained from the interviews and participant quotations, identified by a participant number, are provided to illustrate key concepts.

#### Acceptability

Participants’ perceptions of the workbook’s acceptability were explored during the interviews. A few participants highlighted that the intervention would be more acceptable if introduced earlier in the course of their condition or during the waiting period for a medical treatment. One participant stated, “It would be more useful for someone in the early stages of the condition” (participant 1), while another participant mentioned, “I wish this workbook had been available to me then, because I found it very comprehensive” (participant 5). Additionally, a participant expressed retrospectively, “If I went back to before my treatment, I would have found it a lot more helpful” (participant 2). These findings suggest that the acceptability of the intervention may be influenced by the timing of its implementation.

#### *Practicability*

During the interviews, participants were asked to provide feedback on the practicability of the workbook. Two participants mentioned that the structure and content of the workbook were logically ordered and easy to follow, stating, “It flowed. It flowed for me” (participant 5), and “It’s easy to follow the activities” (participant 2). These comments suggest that the workbook’s layout and design successfully facilitated engagement and adherence to the activities. This feedback indicates that the workbook’s layout and design were well-received, suggesting its practicability in the context of self-directed interventions.

#### Effectiveness

Participants shared their perceptions of the intervention’s potential effectiveness during the interviews. All participants believed the intervention was an effective tool for people living with achalasia, with some participants providing specific reasons for their positive views. For example, one participant commented on the usefulness of signposting to additional resources in the workbook, stating, “Definitely, yeah, most, most definitely. And I think where you’ve signposted at the back, the helpline. I think that’s really invaluable, you know” (participant 1). Another participant highlighted that the content was relatable when they used the intervention, “. I really do because it it’s making you feel understood” (participant 5). Participants also highlighted the positive impact the intervention had on their quality of life, stating, “it gives you confidence” (participant 5) and “It also helps people to live their life to the full” (participant 3)*.* One participant noted that the intervention provided several different ideas and options, which could be revisited in case one approach did not work, “You know you got somebody that’s trying to help you and you think, right, I’ll have to go. If something doesn’t work, you can go back to the workbook because you’ve got several different ideas. So yes, I do think it would help definitely because there’s not a lot of help and advice out there really” (participant 2). These responses suggest that the workbook was perceived as an effective resource for managing achalasia and eating in a social setting.

#### *Affordability*

During the interviews, participants were asked about the affordability of the intervention, specifically the cost of printing and using it. Most participants did not consider the cost of printing to be a major issue, with one participant noting that “I don’t think that’s a problem” (participant 1). However, participants did mention potential barriers, such as not having a working printer and the preference for a shorter workbook with fewer pages. Nevertheless, participants expressed a willingness to pay for the intervention indicating that the affordability of the intervention was not a major concern.

#### Side effects

Participants were also asked about the intervention’s potential negative effects on people who completed it. Four participants were confident that the intervention would not have any negative impact on its users, as it provides them with different strategies and options to try and improve their symptoms. One participant even described it as “all positive” (participant 5). However, one participant was unsure if there could be any negative effects, highlighting the importance of monitoring for any unintended consequences of the intervention. The following quote is from the participant that was not sure about the negative consequences of the workbook: “I can’t speak for others unfortunately, I don’t know. I hope not, I hope” (participant 3). Overall, the lack of concerns raised by participants regarding potential negative effects suggests that the intervention is safe to use.

#### Equity

One participant expressed concerns regarding potential equity issues related to the language and accessibility of the workbook. They highlighted that the language used in the workbook might not be suitable for individuals who are not well-educated or whose first language is not English. Participant 2 commented, “There’s a lot of people who aren’t very well educated or English is not their first language” and “Because when you first learning English, if somebody gave you this book. How many words would you recognise?” This could create barriers for individuals who may not have the same level of literacy or language proficiency as others. Regarding equity, while the intervention shows promise, it is important to note that the inclusion criteria required participants to be able to speak or read English. Therefore, we cannot make definitive claims about its accessibility for non-English speakers.

## Discussion

### Feasibility of recruiting and retaining participants

The current study recruited participants from the Achalasia Action support group through emails that were sent by the moderator of the group. While recruitment in this study using the current strategy gathered sufficient participants, the challenge lay in participant retention and completion rates.

In this study, a retention rate of 50% was achieved which is comparable to the existing literature. For example, this falls within the range observed in a comprehensive literature review of web-based well-being interventions for informal caregivers of people living with dementia. The retention rates in these studies varied between 32.6 and 97.4%, with an average of 70.44%. These figures emphasise the variability in retention rates across different studies and interventions, highlighting the importance of contextual factors in influencing participant retention. [27] The retention rate in this study is too low for a definitive trial and warrants consideration on how to improve retention in future trials. The high rate of loss to follow-up may introduce bias, as those who did not complete the study might have been less engaged with the intervention or experienced poorer outcomes. Since direct follow-up with non-completers was not possible despite multiple contact attempts, arranging a patient group meeting to discuss potential issues and barriers to participation is necessary. This feedback will help identify common challenges, such as difficulty with the intervention content, time constraints, or lack of perceived benefit. Integrating an additional patient group meeting in this study design can improve retention rates and minimise bias.

Establishing a steering group that includes the patient’s voice is critical for enhancing retention in research studies. This group should comprise a diverse mix of patients, researchers, and healthcare providers to ensure a comprehensive understanding of the issues at hand. By including patients who have experienced the study process firsthand, the steering group can provide valuable insights into why participants may be lost to follow-up and what strategies could be effective in mitigating these losses. Their lived experiences and perspectives can uncover barriers that researchers might overlook and help tailor retention strategies that are practical and empathetic. This collaborative approach ensures that the study design, communication, and participant support mechanisms are aligned with the needs and preferences of the participants, ultimately fostering a more participant-centred research environment that is likely to enhance retention rates. Conducting a pilot study with this modification as an interim step may help refine the intervention and ensure its feasibility for a larger, definitive trial. Addressing these points aligns our research approach with the main aim of recruiting and retaining participants, ultimately improving the validity and impact of the intervention study. In a systematic review by Whitaker et al., it was evident that using social media such as Facebook not only aids in efficient recruitment but also fosters a more engaged participant community. This engagement might lead to improved retention and completion rates in studies. Recruiting through Facebook tends to be more efficient than traditional methods such as email or word of mouth. This will give the researcher the ability to connect to harder-to-reach populations. [28] Future studies may benefit from using social media channels such as Facebook groups for people living with achalasia as a way to reach out to a more engaged and active participant pool. This strategy may improve participant involvement and enable a greater understanding of the experiences related to achalasia.

### Acceptability of measures and research procedures

In this study, participants completed a pre-intervention questionnaire and received the co-designed intervention, including activities and techniques. Half of the participants completed the post-intervention questionnaire, and some took part in a short interview. The findings illustrate that the workbook can help some individuals to change their eating behaviour and enhance the social eating experience. Using the APEASE assessment allowed the researchers to evaluate the appropriateness of the intervention. Overall, the participants in this study found the intervention, a workbook for people living with achalasia, to be acceptable, practical, effective, and affordable. They reported that the workbook was easy to follow, comprehensive, and flowed well. Participants believed the intervention could help people living with achalasia by providing them with new strategies and confidence to manage their symptoms. Participants did not report any significant negative side effects from completing the intervention. However, one participant expressed concerns about the suitability of the language used in the workbook for those who are not fluent in English or have lower levels of English literacy.

In the current study, the average age of participants in the completers group was higher than the non-completers group; there were also more women in the completers group. More participants in the completers group reported the use of different interventions before using the one for this study, therefore indicating potential higher self-efficacy. These results are aligned with previous research that reports that older age, higher self-efficacy for the intended health behaviour, and female gender are associated with increased adherence to internet-based interventions. [29–31]

Participants who completed all three stages of this study agreed that the co-designed workbook is an effective tool for building confidence and helping people enhance the social eating experience. These participants also experienced fewer negative symptoms such as pain, stress, and anxiety after completing the intervention. The majority of participants believed that the workbook would be more useful if they received it soon after initially experiencing symptoms of achalasia. However, most of those who completed the workbook successfully expressed their intention to use the workbook and reflect on its contents in the future. The completers group in this study had been living with achalasia for longer than those in the non-completer’s groups. People who had the condition for longer may have had a higher level of readiness for change, and this is in line with the results of a pilot study conducted by Morton et al. Their study shows that there may be a link between changes in risk factors for chronic diseases post-intervention and confounding variables such as self-selection method of participant recruitment. The study shows that people who decided to participate may have been more open to change, and this means that they are more likely to take part in the intervention. [32]

The participants exhibited a slight improvement in their confidence levels regarding eating in social settings following the intervention. This finding suggests a potential positive impact of the intervention on an individual’s confidence. However, as this was a feasibility study with a sample size of 20, the generalisability of these results is limited, and we do not make broader claims about the potential effectiveness of the intervention based on these findings alone. According to research guidelines, sample sizes between 20 and 30 are often recommended for pilot and feasibility studies, as they allow for a manageable yet informative exploration of feasibility without overextending resources. Although small, our sample size aligns with these guidelines and was sufficient to meet the primary objectives of assessing feasibility and gathering preliminary insights into intervention effects. To establish the robustness and generalisability of the intervention’s effects on confidence, future work can involve replicating this study with a larger and more diverse participant cohort. A larger sample size would enhance statistical power, allowing for more reliable conclusions to be drawn regarding the intervention’s ability to facilitate increased confidence in social setting contexts. Moreover, a larger study would permit the exploration of potential moderators or mediators that may influence the relationship between the intervention and confidence outcomes.

Completers of the intervention reported a reduction in their negative symptoms following the completion of the workbook. This finding suggests a potential association between the workbook activities and symptom improvement. However, it is important to acknowledge that various external factors may have influenced these outcomes. Notably, the participants were given flexibility in completing the post-intervention questionnaire, which introduces the possibility of different contextual influences on their responses. Furthermore, the issue of recall bias must be considered, as participants may not accurately remember their symptom experiences in social settings if the questionnaire was not completed immediately after such events. Consequently, caution is needed before drawing definitive conclusions regarding the efficacy of the workbook in positively altering participants’ symptoms. Due to the complexity of each person’s experience and the many ways in which symptoms manifest, it is important to thoroughly investigate circumstances, psychosocial factors, and the participants’ engagement with workbook activities to deeply understand how the intervention may help reduce symptoms. Such research will help us understand the effects of the intervention in a more detailed way and make it easier to facilitate the development of tailored interventions that meet the specific needs of people with achalasia.

A notable observation is that over half of the participants in the completers group had been living with the condition for more than 5 years. Despite their extensive experience with various interventions throughout their journey, these individuals expressed a continued search for tools and strategies to help them in managing their condition. Importantly, participants emphasised the significance of making this co-designed intervention available at the early stages of their diagnosis and treatment pathway. The chronic nature of their condition highlights the persistent challenges faced by people living with achalasia and the ongoing need for effective interventions to alleviate their symptoms and improve their quality of life. The participants’ desire for accessible and timely interventions highlights the importance of early intervention initiatives and the necessity for healthcare providers to provide comprehensive support from the initial stages of diagnosis. These findings emphasise the significance of addressing the needs of people living with achalasia and highlight the potential benefits of readily available interventions integrated into the early stages of their treatment journey. By ensuring the availability of effective interventions and support mechanisms, healthcare providers can contribute to enhancing the overall well-being and long-term outcomes of individuals navigating the challenges associated with achalasia.

### Evaluation of intervention practicability and accessibility challenges

Although most of those who participated and completed all three stages of the study reported that the workbook was beneficial and helped them change their behaviour in a social setting, the assumptions made are limited by the small sample size. Although interviews were arranged soon after the participants completed the post-intervention questionnaire, they were arranged around 2 weeks after they had completed the questionnaire. The time lag between workbook completion, the post-intervention questionnaire, and the interview can affect recall of people’s opinions and perceptions of the workbook.

The intervention may have barriers that widen the gap between participants in terms of access and benefit. One critical issue is that the workbook’s language and design need to be appropriate for a diverse audience to promote equity in access and use. However, the workbook’s content was not assessed for readability, which could contribute to these barriers. Additionally, while the intervention shows promise, the inclusion criteria required participants to speak or read English, based on self-reported capabilities without set criteria for assessment. Consequently, the degree of proficiency and comfort in receiving health information in English were not formally evaluated. This limitation prevents us from making definitive claims about the intervention’s accessibility for non-English speakers and highlights the need for future revisions to ensure the workbook is accessible to all populations.

### Strengths and limitations

In testing the feasibility of the intervention, one of the inherent strengths was patient involvement throughout the research life cycle, including the development of the intervention in the second study. ^(17)^ While the present study primarily focused on implementation rather than design, the collaborative approach ensured that the intervention was initially tailored to meet the specific needs and experiences of those living with achalasia. This tailored approach likely enhanced engagement and feasibility. Participants in the study were members of the Achalasia Action group, which is a forum that provides information and support for people living with achalasia. The involvement of this group facilitated participant engagement and willingness to participate in the intervention. This shared understanding and mutual support within the group likely contributed to the feasibility of the study, especially regarding recruitment and implementation.

For this feasibility study, a priori success criterion was established for the recruitment phase, specifically setting the target to enrol 100% of the desired sample size. This criterion was successfully met, demonstrating the potential effectiveness of the recruitment strategy and confirming sufficient interest among the target population. However, the study did not include predefined criteria for participant retention or adherence. This decision was intentional, as the study aimed to explore these factors to gather insights for future research. While the absence of predefined benchmarks for retention and adherence is acknowledged as a limitation, the data collected provides valuable information for refining these aspects in subsequent trials. Additionally, although the study collected qualitative and quantitative data to evaluate the acceptability of the measures, procedures, and intervention, there were no a priori success criteria set for these components. This exploratory approach has limited the ability to quantitatively assess the success of these elements but has yielded important insights that will inform future studies. While the recruitment target was met, indicating the feasibility of enrolment, the lack of predefined criteria for other feasibility metrics such as retention and adherence is recognised as a limitation. Future research will benefit from establishing more comprehensive a priori criteria to guide feasibility assessments more rigorously.

The study acknowledges that the sample size was small, which is a characteristic typical of feasibility studies. It is important to note that this study is designed to assess the feasibility of recruitment and implementing a co-designed intervention rather than drawing conclusions on its efficacy. Although this is understandable given the rare nature of achalasia, a small sample size limits the generalisability of the findings. The relatively small sample size results in a wide confidence interval when estimating retention rates, which reduces the precision and reliability of these estimates for planning future studies. Additionally, estimating standard deviation with a sample of this size provides only a preliminary indication of variability, and may not accurately reflect the full range of potential outcomes in a larger population. These constraints underscore the exploratory nature of this study, and therefore, caution should be exercised in generalising the results to the wider population of people living with achalasia or those participating in support groups.

Future trials should aim to include larger and more diverse participant cohorts to strengthen the robustness of conclusions. Since the participants were all members of the Achalasia Action group, they shared similar experiences and characteristics related to their condition. This homogeneity may limit the diversity and variability of perspectives and behaviours within the study, potentially affecting the generalisability of the findings to a broader population of people living with achalasia. Future research should seek to include participants from diverse backgrounds to ensure more representative results. The study did not include a control group for comparison. Without a control group, it is challenging to determine the extent to which the co-designed intervention specifically contributed to changes in eating behaviours, as other factors, such as external influences, may have influenced the outcomes. The absence of a control group limits the ability to establish a causal relationship between the intervention and the observed changes. Including a control group in future trials would enable better comparison and interpretation of intervention effects, strengthening the validity of conclusions. The study relied on self-report measures to assess changes in eating behaviours. Self-reported data are subject to recall biases, social desirability biases, and individual interpretations. Future research should consider incorporating objective measures or validated instruments to enhance the accuracy and reliability of outcome assessments. These limitations may affect the accuracy and reliability of the reported outcomes, potentially compromising the validity of the findings.

### Future work

The findings from our current study serve as a foundation for shaping the direction of future research. Conducting a study with a larger sample size would provide more robust and representative findings. While we could not draw any conclusions with regard to change between the two groups of completers and non-completers, a larger sample will allow the researcher to undertake statistical analysis and identify the effectiveness of the intervention. On the other hand, while the current study focused on participants from the Achalasia Action group, future research could aim to include participants with diverse demographic characteristics, such as age, gender, and cultural backgrounds. This would allow for a more comprehensive understanding of the feasibility and potential effectiveness of the co-designed intervention across different populations. Employing a randomised controlled design would strengthen the study’s ability to establish causal relationships between the intervention and changes in eating behaviours. By randomly assigning participants to an intervention group or a control group, researchers can more confidently attribute the observed effects to the intervention itself. Assessing the sustainability of changes in eating behaviours over an extended period would provide valuable insights into the long-term effectiveness of the co-designed intervention. Conducting follow-up assessments at multiple time points after the completion of the intervention can help determine whether the observed changes are maintained or diminish over time. Moreover, the feedback received on the intervention’s design and delivery allows the researcher to refine the intervention along with refining recruitment with a keen focus on optimising its efficacy in changing eating behaviours and supporting eating in social settings for individuals living with achalasia. Future studies should also focus on improving retention rates by implementing targeted engagement strategies, such as personalised follow-up communications. Additionally, qualitative research should be conducted to clearly identify and address the barriers and facilitators that influence participant adherence in achalasia interventions. By addressing these areas in future studies, researchers can further enhance the understanding of the effectiveness of this co-designed intervention to change eating behaviour in social settings for people living with achalasia.

## Conclusions

The study recruited to the target sample and retained half of the participants at follow-up, indicating the feasibility of engaging people living with achalasia in the intervention. However, future work is needed to improve the retention rate. The recruitment methods utilised resulted in a rate that appears to be sufficient for scaling this trial, but reconsideration may be necessary to ensure a sample that is more representative of the broader achalasia population including diverse age groups, socioeconomic backgrounds, and ethnicities. This study has also provided valuable insights into the feasibility of the recruitment and retention methods, suggesting that with some adjustments, these methods could be effectively used in future trials. The intervention demonstrated usability and acceptability, as participants actively engaged and found it valuable. Participants reported positive experiences, suggesting potential effectiveness in changing eating behaviours in social settings. Moreover, the use of the APEASE criteria for the intervention’s evaluation offered valuable insights into its implementation. Additionally, the co-designed approach allowed for participants’ active involvement, which likely increased the intervention’s relevance and potential effectiveness. Despite the limitations, such as the small sample size, the study provides valuable insights into the feasibility of the recruitment and acceptability of the co-designed intervention. Overall, this study serves as a foundation for future research to pilot this intervention on a larger scale to change eating behaviours and improve the quality of life for people living with achalasia.

The findings of this feasibility study have important implications for both the field of achalasia research and the lives of people living with this condition. By exploring the feasibility of implementing a co-designed intervention targeting challenging eating behaviours in social settings, this study introduces a new approach to address the specific needs of people living with achalasia. This study contributes to the existing literature by shedding light on the potential feasibility and practical considerations associated with the intervention used in this study. Ultimately, the successful development and implementation of tailored interventions have the potential to significantly enhance the quality of life for people with achalasia, empowering them to navigate social eating situations with greater confidence and improved outcomes.

## Data Availability

The datasets generated and/or analysed during the current study are not publicly available because participants can be identified from them, but the anonymised data are available from the corresponding author upon reasonable request.

## Abbreviations

COM-B model: Capability, Opportunity, and Motivation-Behaviour model
APEASE: Affordability, Practicality, Effectiveness and cost-effectiveness, Acceptability, Side effects/safety, and Equity
LOS: Lower oesophageal sphincter
BCT: Behaviour change techniques
TDF: Theoretical Domains Framework
BCW: Behaviour change wheel
SD: Standard deviations
